# Changes in household purchasing of soft drinks following the UK soft drinks industry levy by household income and composition: controlled interrupted time series analysis, March 2014 to November 2019

**DOI:** 10.1136/bmjnph-2024-000981

**Published:** 2025-01-16

**Authors:** Nina Trivedy Rogers, Steven Cummins, David Pell, Harry Rutter, Stephen J Sharp, Richard D Smith, Martin White, Jean Adams

**Affiliations:** 1 Department of Public Health, Environments and Society, London School of Hygiene & Tropical Medicine, London, UK; 2 MRC Epidemiology Unit, Institute of Metabolic Science, University of Cambridge, Cambridge, UK; 3 Department of Social and Policy Sciences, University of Bath, Bath, UK; 4 Faculty of Health and Life Sciences, University of Exeter, Exeter, UK

**Keywords:** Nutrition assessment, Nutritional treatment, Dietary patterns

## Abstract

**Background:**

The WHO recommends taxes on sugar sweetened beverages (SSBs) to improve population health. We examined changes in volume of and amount of sugar in purchases of soft drinks according to household income and composition, 19 months following the implementation of the UK soft drinks industry levy.

**Methods:**

Data were from the Kantar Fast Moving Consumer Goods panel, a market research panel which collects data on weekly household purchases (mean weekly number of households=21 908), March 2014–November 2019. Interrupted time series analysis of volume and sugar purchases was used to estimate absolute and relative differences in the volume and amount of sugar in soft drinks, confectionery and alcohol purchased weekly by household income (<£20 000, £20–50 000 or >£50 000) and composition (presence of children (<16 years) in the household (yes or no)), 19 months after soft drinks industry levy (SDIL) implementation, compared with the counterfactual scenario based on pre-announcement trends and using a control group (toiletries).

**Results:**

By November 2019, purchased weekly sugar in soft drinks fell by 7.46 g (95% CI: 12.05, 2.87) per household but volumes of drinks purchased remained unchanged, compared with the counterfactual. In low-income households, weekly sugar purchased in soft drinks decreased by 14.0% (95% CI: 12.1, 15.9) compared with the counterfactual but in high-income households increased by 3.4% (1.07, 5.75). Among households with children, sugar purchased decreased by 13.7% (12.1, 15.3) but increased in households without children by 5.0% (3.0, 7.0). Low-income households and those with children also reduced their weekly volume of soft drinks purchased by 5.7% (3.7, 7.7) and 8.5% (6.8, 10.2) respectively. There was no evidence of substitution to confectionary or alcohol.

**Conclusion:**

In the second year following implementation of the SDIL, effects on sugar purchased were greatest in those with the highest pre-SDIL purchasing levels (low-income households and those with children). The SDIL may contribute to reducing dietary inequalities.

**Trial registration number:**

ISRCTN18042742. Registered: August 2017.

**WHAT IS ALREADY KNOWN ON THIS TOPIC**

- The WHO recommends taxes on sugar sweetened beverages (SSBs) to improve population health; evidence indicates these can successfully reduce population purchasing and consumption but differential impacts across demographic groups have been less studied.

**WHAT THIS STUDY ADDS**

- Households with the lowest incomes (<£20 000/year) and with children living in them had the largest reductions in purchases of sugar from soft drinks, 19 months after the implementation of the UK soft drinks industry levy.
- Sugar purchased by households with the lowest incomes dropped by ~70 g of sugar per household per week, equivalent to just over two 250 mL servings of a drink containing 5 g sugar per 100 mL per person per week. Households with children living in them reduced their purchasing of sugar from soft drinks by 56 g per household per week.

**HOW THIS STUDY MIGHT AFFECT RESEARCH, PRACTICE OR POLICY**

- These findings suggest that the UK soft drinks industry levy may contribute to reducing existing inequalities in dietary intake with strongest benefits in households with the lowest incomes and those with children.

## Background

 Consumption of sugar sweetened beverages (SSBs) is associated with poor health outcomes including non-communicable diseases such as cardiovascular disease, type II diabetes and obesity.^1^ There are inequalities in consumption of SSBs with lower socioeconomic groups consuming more.^2^ High intake of SSBs is also common among children and adolescents and is linked to overweight and obesity in this age group.^3^

The introduction of SSB taxes in a number of countries has been seen as largely successful as a measure to support reductions in dietary intake of added sugar via SSBs.^4^ Indeed, the WHO has recommended taxation of SSBs to reduce consumption of added sugars and improve health.^5^ In response to the UK childhood obesity crisis, the UK soft drinks industry levy (SDIL) on manufacturers, importers and bottlers of soft drinks was announced in March 2016 and implemented in April 2018.^6^ This differed from most other SSB taxes as its primary aim was to reduce sugar consumption by incentivising reformulation rather than to pass higher prices of soft drinks to consumers.^7^ The SDIL was designed as a two-tiered levy with a higher tier for drinks containing over 8 g of sugar per 100 mL (levied at a rate of £0.24 per litre) and a lower tier for drinks containing 5–8 g of sugar per 100 mL (levied at a rate of £0.18 per litre).^6^ Drinks with less than 5 g sugar per 100 mL are not levied.^6^ A number of categories are exempted and not levied irrespective of sugar content, for example, no-added-sugar fruit juices, milk-based drinks, and drinks sold as powder. Companies manufacturing less than one million litres/year are also exempt. One year after the implementation of the UK SDIL, households in the UK were purchasing 2.7% less sugar from take-home drinks (accounting for pre-announcement trends) while the volumes purchased had increased by 2.6%,^8^ suggesting reformulation of SSBs had occurred. This finding has been reinforced by a previous study that examined the sugar content of drinks available in UK supermarkets from 1 year prior to the announcement of SDIL to 1 year post-implementation of SDIL and found that soft drinks with sugar concentration over 5 g/100 mL fell by approximately 34 percentage points suggesting that the levy incentivised manufacturers to reformulate soft drinks and reduce their sugar content.^9^

However, while evidence suggests that SSB taxes have been effective at reducing sales and dietary intake of added sugar from SSBs, it is uncertain whether they reduce inequalities in sugar consumption from SSBs.^4^ In the UK, no study has examined the effect of the SDIL across sociodemographic groups. This is an important gap because in high-income countries, such as the UK, the burden of obesity and other diet-related non-communicable diseases disproportionately affects those with lower income^10^ and those living in deprived neighbourhoods.^11^ Children have been identified as a particularly important target population for obesity prevention measures. While microsimulation modelling studies have projected similar health benefits across socioeconomic groups^12 13^ or greater health benefits for health in lower income groups,^14^,^16^ only a few real-world studies have studied these effects. These report mixed findings. In Chile, Catalonia and Philadelphia, higher socioeconomic groups were more responsive to SSB taxes.^17^,^19^ However, in Mexico, Tonga and elsewhere in the USA, lower socioeconomic groups were more responsive.^20^,^22^ These differences in response to SSB taxes across socioeconomic groups might reflect the structure of differing SSB taxes, different background contexts as well as differences in particular outcomes studied—including sales, purchasing and expenditure. Fewer studies have explored differences in the effect of SSB taxes by household composition^23^ but one study from Mexico found greater impacts in households with children than without.^21^

There is some evidence on the longer-term (>12 months) impacts of SSB taxes on purchasing and consumption of soft drinks. An evaluation of SSB taxes in five large cities in the USA found that 2 years after implementation of the tax, there was a 33% reduction in the volume of soft drinks purchased.^24^ In addition, sustained reductions in purchases of SSBs have been observed in Mexico 2 years after the tax was implemented and compared with pre-tax trends^25^ with a suggestion of some plateauing in purchasing by the third year.^26^

To add to this evolving literature, we examined whether purchased sugar in soft drinks and the purchased volume of soft drinks changed following the announcement and implementation of SDIL. We examined this overall and according to household income levels and in households with and without children. We also investigate if there is any evidence of substitution occurring by examining changes in purchases of sugar from confectionery or volume of alcohol.

## Methods

### Study timeline

Controlled interrupted time series (CITS) analysis was used to compare changes in the amount of sugar in, and volume of, purchased soft drinks bought for consumption in the home, with the counterfactual scenario in which neither the announcement nor implementation of the SDIL happened. The CITS ran from week 1 in March 2014, through the time of the SDIL announcement (March 2016; study week 108), and the SDIL implementation^27^ (April 2018; study week 214) until its final week in November 2019 (study week 295).

### Data source

We used data from Kantar Fast Moving Consumer Goods (KFMCG) panel, a market research company which collects household panel data on purchases of food, drink and other items from households in Great Britain (thus excluding Northern Ireland). KFMCG provided household purchasing data at the weekly level. The weekly mean number of households was 21 908. Households recruited into the panel are given a handheld scanner to record the barcodes of purchased items brought into the home and a book of barcodes to record unpackaged items. The information (including online sales and deliveries) is uploaded and sent to KFMCG who link the purchasing information to nutritional data on a continual basis. Households record and update their demographic characteristics annually and as an incentive for taking part, they receive gift vouchers equivalent to £100 ($122; €112) annually. KFMCG excludes households that record fewer than six purchases weekly along with those whose adjusted weekly spend is lower than an undisclosed minimum.

### Product categories

Purchased soft drinks considered in the study included both levy-liable and levy-exempt types that were purchased and brought into the home. Inclusion of both levy-exempt and levy-liable soft drinks in the study enabled examination of the full impact of the SDIL on all soft drink purchases and captures potential soft drink products that may have been used as substitutes but not otherwise included if levy-exempt soft drinks were not considered in the analysis. In sensitivity analyses, purchases of alcohol (including alcoholic and alcohol replacement drinks) and confectionery (sugar and chocolate confectionery) were explored separately to determine whether any reductions in sugar from, or volumes of, soft drink purchases were substituted by increases in purchasing of alcohol or sugar from confectionery. To account for background trends in household purchases, toiletries (shampoo, hair conditioner and liquid soap) were incorporated as a non-equivalent control category. Toiletries make a suitable control for a CITS because we do not consider them to be directly or indirectly affected by the UK SDIL, their purchasing is unaffected by seasonality and are likely to have similar purchase volumes by households irrespective of socioeconomic position and other confounders.

### Household demographics

Total gross household income was categorised into three groups, less than £20 000 (low), £20 000–49 999 (middle) and £50 000 and over (high). Median annual household income in the UK in 2019 was estimated to be ~£45 000.^28^ Households were categorised into those with children (aged less than 16 years) present and those without.

### Statistical analysis

Prior to analysis, products were assigned to the SDIL relevant groups (eg, all soft drinks, alcohol, confectionery and toiletries) based on product groups assigned by KFMCG and product names. Analysis was based on weekly lists of purchasing by product line, which report the type of purchase, sugar content (per 100 g/mL) and volume or mass purchased. Proprietary grossing up weights, created by Kantar Worldpanel, were used throughout our analysis to extrapolate from the size of the panel to the size of the population in Great Britain (GB) and to ensure the sociodemographic spread of the panel was representative of the GB population. Weekly household sugar purchases were calculated as sum of all (sugar concentration × volume × KWP weight)/number of households. In subgroup analysis, weekly purchasing within a demographic group was further adjusted by multiplying it by the proportions of households from the population of Great Britain that were in each demographic group.^29 30^

CITS was performed using a controlled generalised least squares model with an autocorrelation-moving average (ARMA) correlation structure where the autoregressive order (p) and moving average order (q) were selected to minimise the Akaike Information Criterion (AIC) value of the model. All models included adjustment for mean monthly temperature and the months of December and January, since purchasing of soft drinks is influenced by seasonal factors (see online supplemental material). Predicted counterfactual values (assuming the SDIL had neither been announced nor implemented) were calculated from the model. The difference in weight or volume between the observed and counterfactual values was estimated at week 295 (03/11/2019) and expressed in absolute grams or millilitres, respectively, and as a percentage. CIs in this study were calculated from standard errors estimated using the delta method. Analysis was conducted in R V.4.1.0.

### Changes to Protocol

Three changes were made to the published protocol.^31^ First, KFMCG provided weekly rather than monthly purchasing data which allowed us to improve the precision of our findings. Second, we initially proposed the CITS to finish in March 2020, 2 years after SDIL was implemented. However, because of potential household stockpiling of grocery products in anticipation of (i) the UK leaving the European Union in December 2019 and (ii) national lockdown due to the COVID-19 pandemic,^27^ follow-up was ended in November 2019. Third, to examine disparities across socioeconomic groups, socioeconomic position was operationalised as household income, which was considered a stronger indicator of material living standards, compared with social class of the main household member.

### Patient and public involvement

A steering group, including two lay members, meet twice a year to discuss the broader issues around SDIL evaluation. The public and participants were not involved in developing the research question or other aspects of the design reported here.

## Results

Table 1 summarises the mean volume and weight of sugar in drinks purchased per household in the week prior to the SDIL announcement and the week prior to its implementation, and in the final week of follow-up (19 months post-implementation) in the total population, by income group and households with and without children. In all sociodemographic groups, average weekly sugar purchased in drinks reduced over the study period. In the week prior to the announcement, households in the lowest income group purchased nearly twice as much sugar from, and volume of, soft drinks than mid-income households and approximately four-times more sugar from, and volume of, soft drinks than households in the highest income groups. Households with children purchased approximately 40% more sugar and 30% higher volume of soft drinks than households without children.

**Table 1:** Mean weight of sugar in, and volume of purchased soft drinks per household per week in the week prior to announcement, implementation and 19 months post-implementation of the UK soft drinks industry levy

Sociodemographic characteristics	Population %	Mean volume (mL) of, and weight of sugar (g) in purchased soft drinks per household per week		
		1 week prior to announcement	1 week prior to implementation	19 months post-implementation
Weekly weight of sugar (g) (SD)				
Total population		363.5 (17.1)	336.7 (23.6)	308.4 (18.9)
Income				
<20 000	21	627.6 (29.3)	565.9 (38.8)	472.3 (30.7)
20 000–50 000	59	315.8 (15.5)	301.7 (21.7)	280.1 (17.2)
>50 000	20	157.9 (9.80)	146.9 (13.5)	134.1 (9.34)
Children in household				
Yes	28	453.0 (25.14)	428.78 (35.36)	371.84 (25.85)
No	72	328.8 (16.9)	300.9 (20.3)	273.0 (16.20)
Weekly volume of drinks (mL) (SD)				
Total population		7595.2 (295.3)	7547.5 (466.1)	7779.0 (465.5)
Income				
<20 000	21	12 747.3 (530.8)	12 263.4 (761.1)	11 908.5 (738.6)
20 000–50 000	59	6659.1 (275.5)	6849.8 (438.0)	7334.9 (456.2)
>50 000	20	3472.4 (170.4)	3500.0 (257.1)	3608.1 (240.6)
Children in household				
Yes	28	9220.9 (450.9)	9401.6 (671.8)	9536.9 (627.7)
No	72	6963.0 (305.2)	6826.4 (417.6)	7095.4 (441.2)

Unless stated otherwise, all estimates below are per household per week, with respect to the counterfactual scenario (estimated from modelled pre-announcement trends (weeks 1–108) at 19 months post-implementation (November 2019 or time point week 295)).

### Changes in amount of sugar from purchased soft drinks by household income and composition

**Figure 1:**
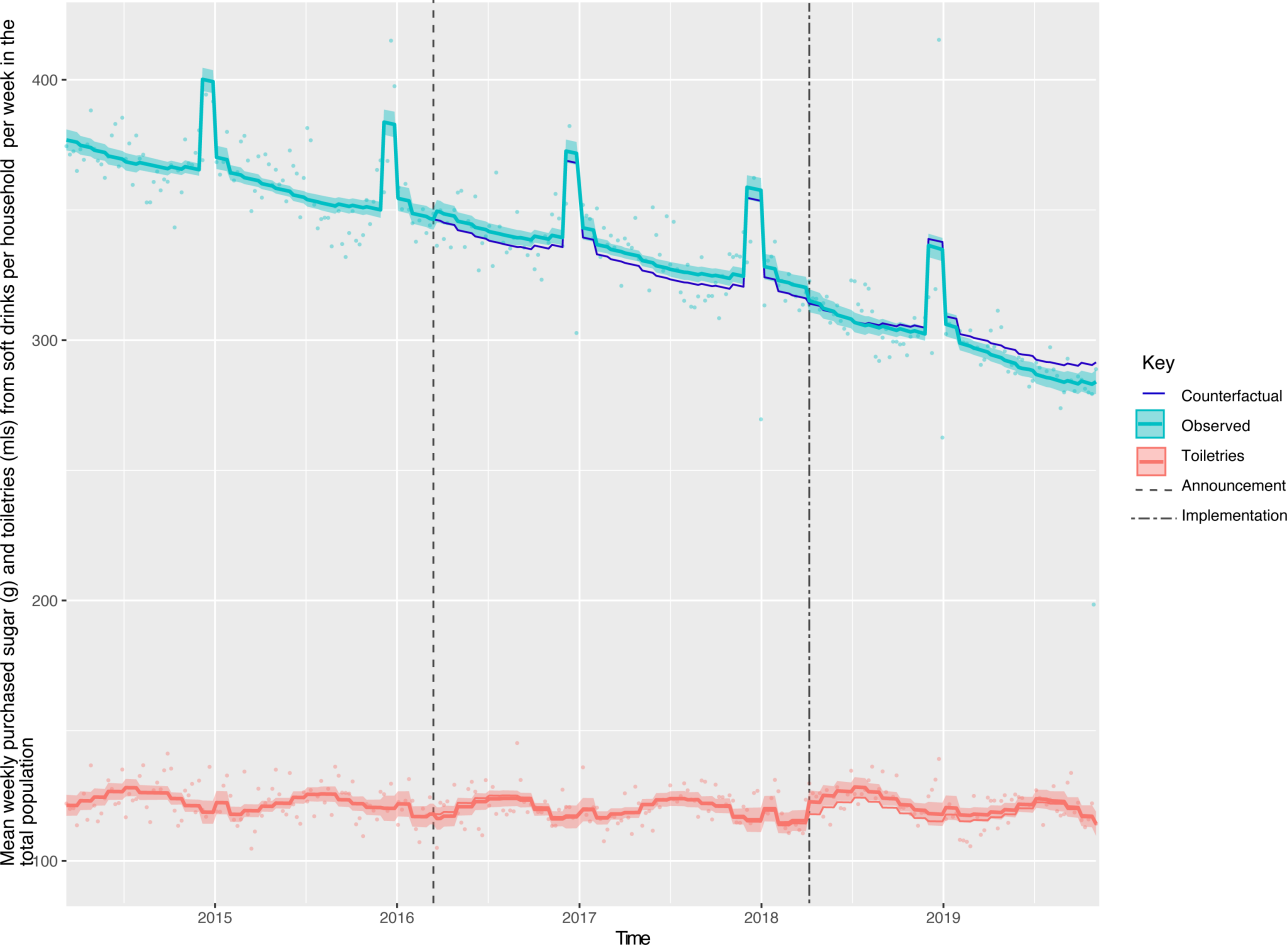
Weight (**g**) of sugar from soft drink products purchased per household per week in the total population, from March 2014 to November 2019. Observed and modelled amounts of sugar in all soft drinks (drinks liable to the SDIL and non-liable drinks). Light blue points show observed data and light blue lines (with light blue shadows) show modelled data (and 95% CIs) of sugar from purchased soft drinks. The dark blue line indicates the counterfactual line based on pre-announcement trends and had the announcement and implementation not happened. The red line (and shadow) indicates modelled toiletries (control group). The first and second dashed lines indicate the announcement and implementation of SDIL, respectively. SDIL, soft drinks industry levy.

**Figure 2:**
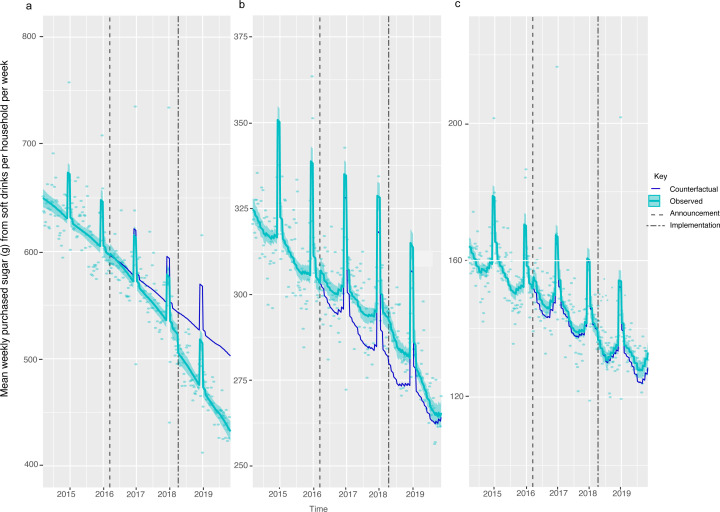
Weight (g) of sugar from soft drink products purchased per household per week, by gross household income levels, from March 2014 to November 2019. Observed and modelled amounts of sugar in all soft drinks (drinks liable to the SDIL and non-liable drinks) by annual gross household income levels of (a) <20 000, (b) £20 000–£50 000 and (c) £50 000 or more. Light blue points show observed data and light blue lines (with light blue shadows) show modelled data (and 95% CIs) of sugar from purchased soft drinks. The dark blue line indicates the counterfactual line based on pre-announcement trends and had the announcement and implementation not happened. The first and second dashed lines indicate the announcement and implementation of SDIL, respectively. The scales on the Y axis vary between panels and modelled toiletries have been removed (see online supplemental figure S1 for inclusion of toiletries) to maximise the resolution of the graphs. SDIL, soft drinks industry levy.

**Figure 3:**
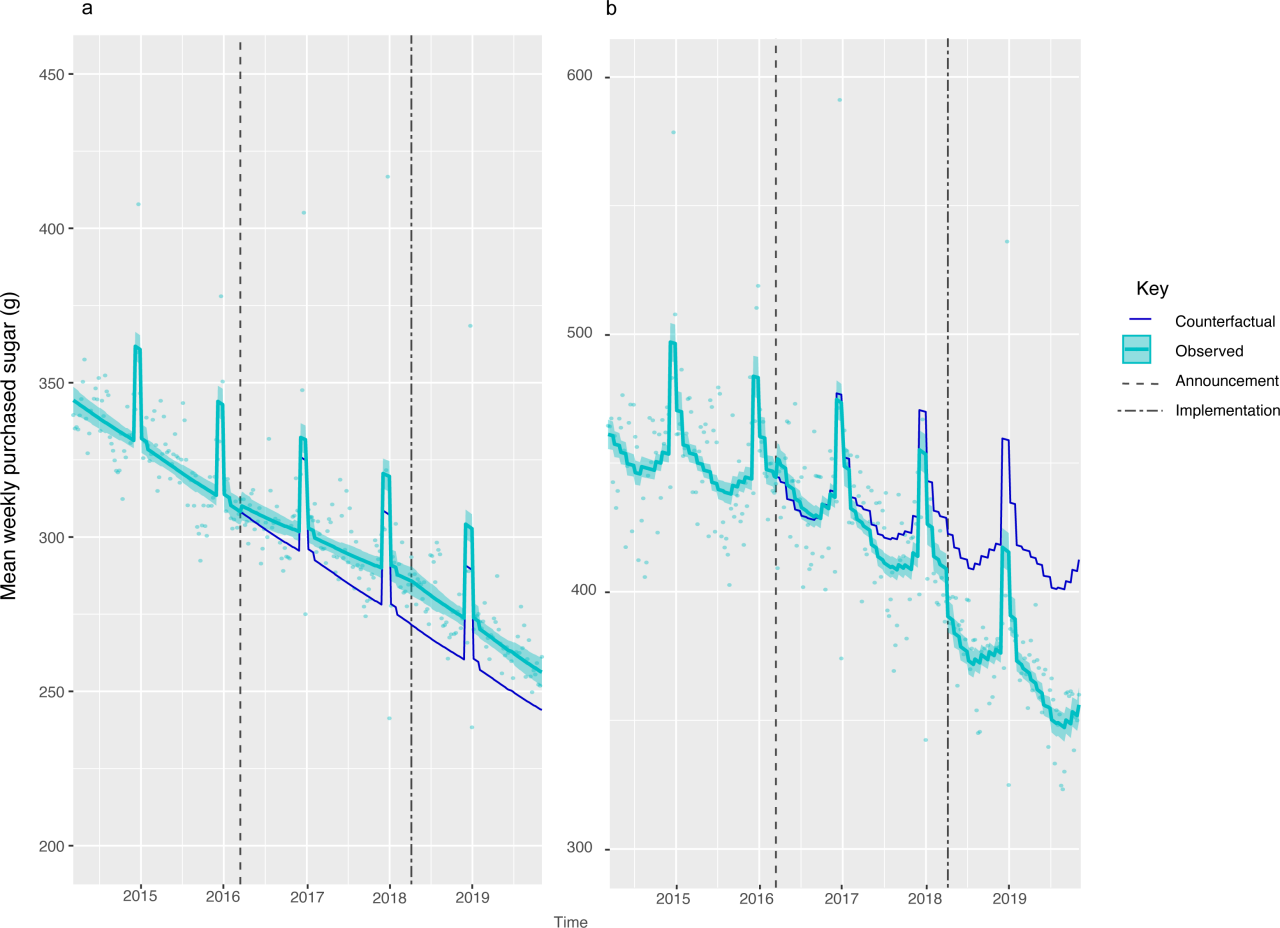
Weight (g) of sugar from soft drink products purchased per household per week, by whether households have children or not, from March 2014 to November 2019. Observed and modelled amounts of sugar in all soft drinks (drinks liable to the SDIL and non-liable drinks) by (a) households with no children and (b) households with children (<16 years). Light blue points show observed data and light blue lines (with light blue shadows) show modelled data (and 95% CIs) of sugar from purchased soft drinks. The dark blue line indicates the counterfactual line based on pre-announcement trends and had the announcement and implementation not happened. The first and second dashed lines indicate the announcement and implementation of SDIL, respectively. The scales on the Y axis vary between panels and modelled toiletries have been removed to maximise the resolution of the graphs. SDIL, soft drinks industry levy.

Across all households in GB, there was a 7.46 g (95% CI: 2.87, 12.05) or 2.56% (95% CI: 0.62, 4.49)) reduction in weight of sugar purchased from soft drinks (figure 1, table 2). The largest reduction was observed in the lowest income households (figure 2, online supplemental figure S1) and households with children (figure 3, online supplemental figure S2). Small increases in sugar purchased from soft drinks were seen in high-income households and households without children. The sugar purchased from soft drinks was 70.27 g (60.63, 79.91) or 13.98% (12.07, 15.9) lower in low-income households per household per week and 56.39 g (49.82, 62.97) or 13.67% (12.08, 15.27) lower in households with children at 19 months post-implementation compared with the counterfactual. Purchased sugar from soft drinks was 4.38 g (1.37, 7.39) or 3.41% (1.07, 5.75) higher in high-income households and 12.2 g (7.29, 17.18) or 5.01% (2.99, 7.04) higher in households with no children present, respectively. Sugar purchased via soft drinks in middle-income households remained unchanged.

**Table 2:** Absolute and relative changes in volume of, and weight of sugar in soft drinks purchased per household per week, compared with the counterfactual estimated from pre-announcement trends, at 19 months post-implementation of the UK soft drinks industry levy

	Pre-announcement to post-implementation of SDIL	
	Absolute (g per household per week)	Relative (%)
**Sugar (g)**		
Total population	−7.46 (−12.05, –2.87)	−2.56 (−4.49, –0.62)
Income		
<20 000	−70.27 (−79.91, –60.63)	−13.98 (−15.90, –12.07)
20 000–50 000	1.30 (−2.48, 5.08)	0.49 (−0.94, 1.92)
>50 000	4.38 (1.37, 7.39)	3.41 (1.07, 5.75)
Children		
Yes	−56.39 (−62.97, –49.82)	−13.67 (−12.08, –15.27)
No	12.2 (7.29, 17.18)	5.01 (2.99, 7.04)
**Volume (mL)**		
Total population	124.5 (−7.64, 256.71)	1.71 (−0.10, 3.52)
Income		
<20 000	−674.8 (−907.5, –442.1)	−5.74 (−7.72, –3.76)
20 000–50 000	245.2 (126.2, 364.1)	3.61 (1.86, 5.36)
>50 000	53.3 (−16.5, 123.0)	1.54 (−0.48, 3.55)
Children		
Yes	−849.37 (−1020.58, –678.16)	−8.50 (−10.22, –6.79)
No	540.93 (401.79, 680.06)	8.71 (6.47, 10.95)

SDIL, soft drinks industry levy.

### Changes in volume of purchased soft drinks by household income and composition

**Figure 4:**
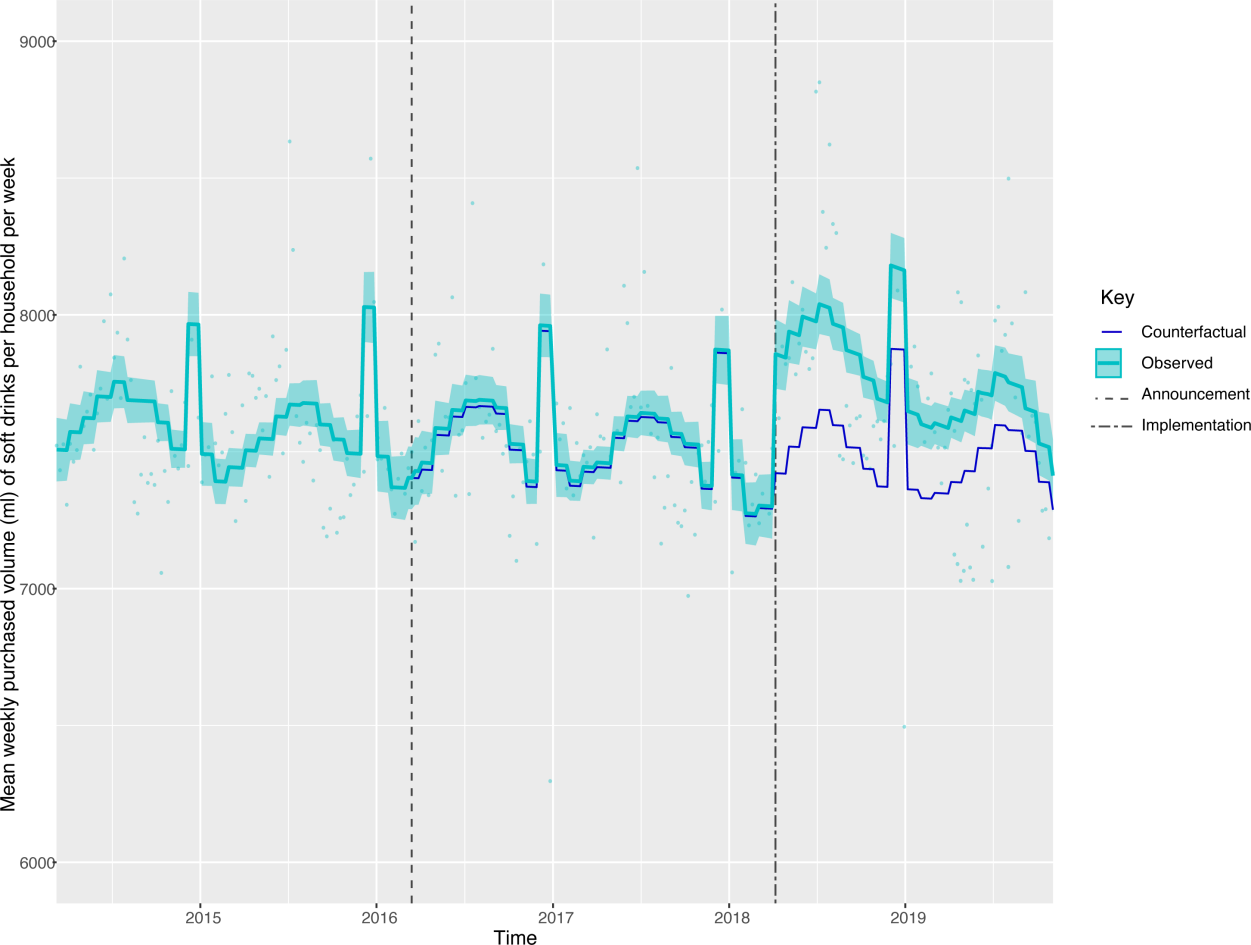
Volume (mL) of soft drink products purchased per household per week in the total population, from March 2014 to November 2019. Observed and modelled volumes of all soft drinks (drinks liable to the SDIL and non-liable drinks). Light blue points show observed data and light blue lines (with light blue shadows) show modelled data (and 95% CIs) of volumes of purchased soft drinks. The dark blue line indicates the counterfactual line based on pre-announcement trends and had the announcement and implementation not happened. The first and second dashed lines indicate the announcement and implementation of SDIL, respectively. Modelled toiletries have been removed to maximise the resolution of the graphs. SDIL, soft drinks industry levy.

**Figure 5:**
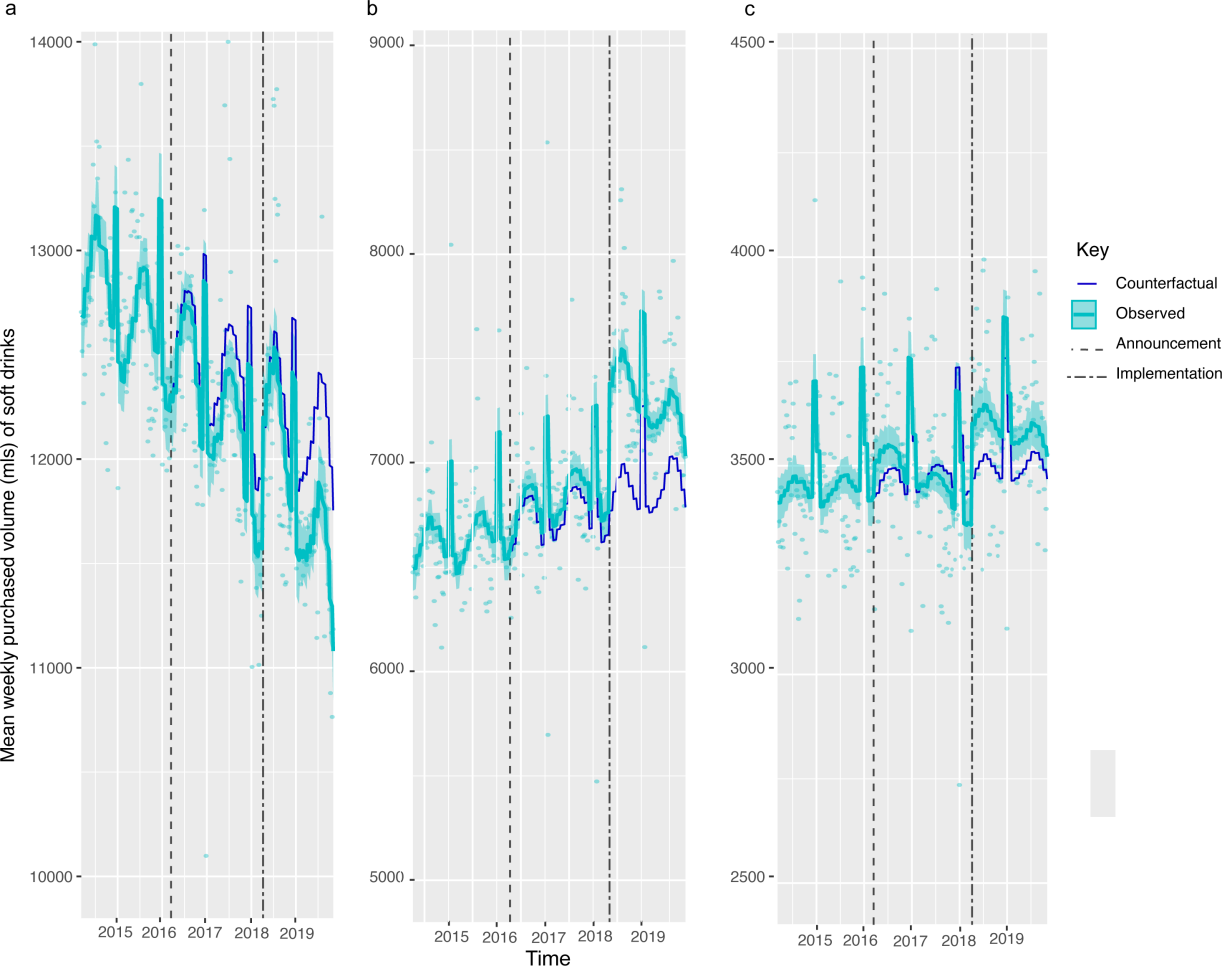
Volume (mL) of soft drink products purchased per household per week, by gross household income levels, from March 2014 to November 2019. Observed and modelled volumes of soft drinks (drinks liable to the SDIL and non-liable drinks) by annual gross household income levels of (a) <20 000, (b) £20 000–£50 000 and (c) £50 000 or more. Light blue points show observed data and light blue lines (with light blue shadows) show modelled data (and 95% CIs) of sugar from purchased soft drinks. The dark blue line indicates the counterfactual line based on pre-announcement trends and had the announcement and implementation not happened. The first and second dashed lines indicate the announcement and implementation of SDIL, respectively. The scales on the Y axis vary between panels and modelled toiletries have been removed to maximise the resolution of the graphs. SDIL, soft drinks industry levy.

**Figure 6:**
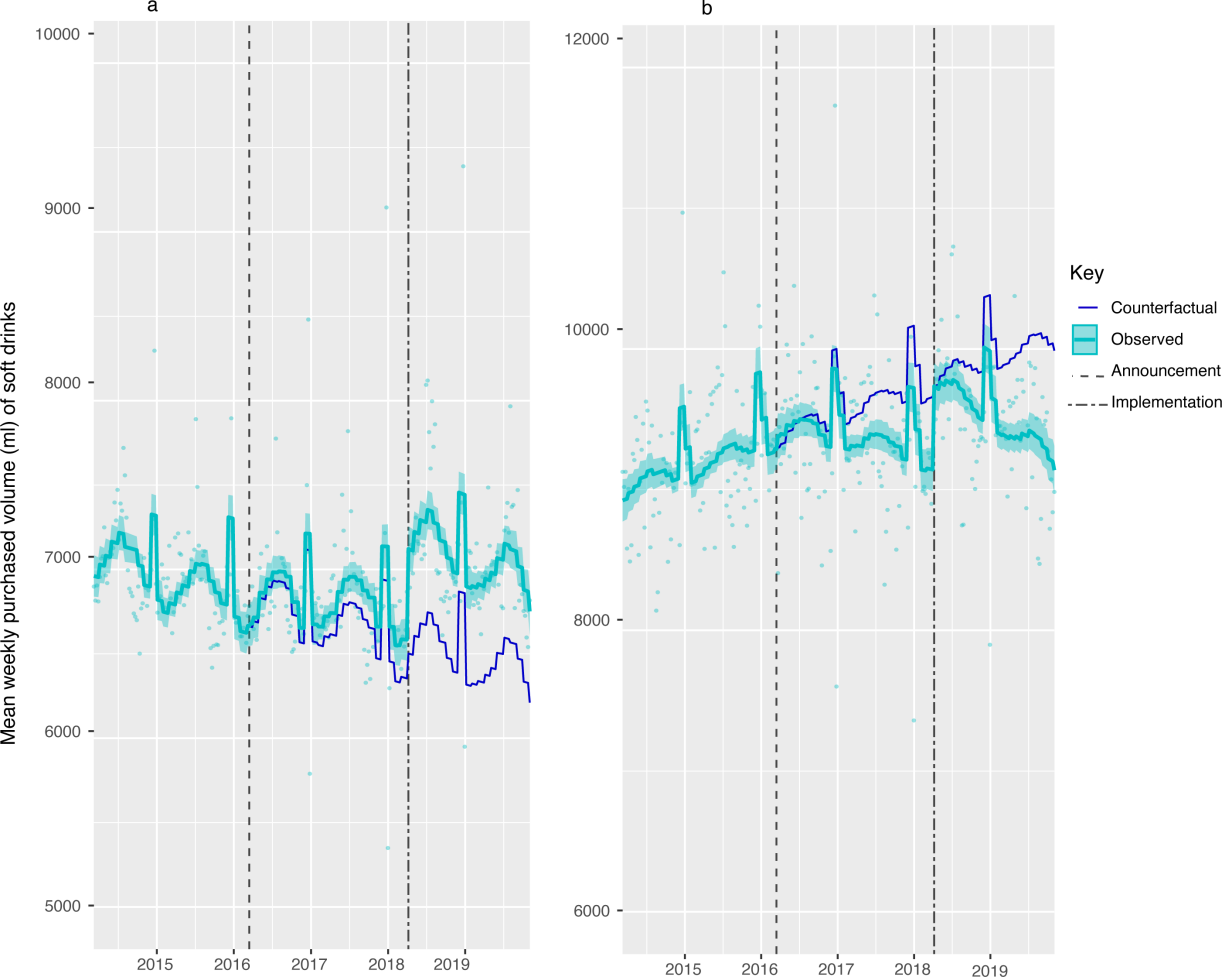
Volume (mL) of soft drink products purchased per household per week, by whether households have children or not, from March 2014 to November 2019. Observed and modelled volumes of soft drinks (drinks liable to the SDIL and non-liable drinks) by (a) households with no children and (b) households with children (<16 years). Light blue points show observed data and light blue lines (with light blue shadows) show modelled data (and 95% CIs) of sugar from purchased soft drinks. The dark blue line indicates the counterfactual line based on pre-announcement trends and had the announcement and implementation not happened. The first and second dashed lines indicate the announcement and implementation of SDIL, respectively. The scales on the Y axis vary between panels and modelled toiletries have been removed to maximise the resolution of the graphs. SDIL, soft drinks industry levy.

Compared with the counterfactual, at 19 months post-implementation, there was no overall change in the volume of soft drinks purchased across all households (figure 4, table 2). However, there were reductions in the volumes of drinks purchased by the lowest income households (figure 5) and those with children (figure 6), with increases in middle-income households and households without children. The volume of drinks purchased was 674.8 mL (442.1, 907.5) or 5.74% (3.76, 7.72) lower per week in low-income households and 849.37 mL (678.16, 1020.58) or 8.50% (10.22, 6.79) lower per week in households with children. In middle-income households and households without children, the volume of drinks purchased per week was higher by 245.2 mL (126.2, 364.1) or 3.61% (1.86, 5.36) and 540.93 mL (680.06, 401.79) or 8.71% (6.47, 10.95), respectively.

### Purchasing of sugar through confectionery

Purchasing of sugar via confectionery was unchanged across all household income groups (online supplemental figure S3) and households with and without children (online supplemental figure S4), compared with the counterfactual at 19 months post-implementation (online supplemental table S1).

### Purchasing of alcohol

Compared with the counterfactual estimated from pre-announcement trends, the volume of purchased alcohol reduced (p≤0.05) overall and across all income groups (online supplemental figure S5) and in households with children (online supplemental figure S6). In households without children, the volume of alcohol increased (p≤0.05) compared with the predicted counterfactual (online supplemental table S2).

## Discussion

### Summary of principal findings

This is the first analysis to examine differential impacts of the SDIL on changes in sugar from, and volume of, purchased soft drinks, and according to household income and composition. At 19 months post-implementation, sugar purchased from soft drinks fell overall (by 8 g per household per week, or 3%) compared with the counterfactual of no intervention, but volume did not. Alongside, we found evidence that the SDIL reduced inequalities in sugar purchasing associated with soft drinks. Lower income households, and those with children, purchased the most sugar from, and volume of, soft drinks at baseline. They also had the largest reductions in sugar from (by 70 g per household per week or 14% in the lowest income households and 56 g or 14% in households with children), and volume of (by 675 mL or 6%, and 849 mL or 9% respectively), soft drinks purchased following the announcement and implementation of the UK SDIL. A 70 g reduction in sugar per household per week is equivalent to just over two 250 mL servings of a lower-levy tier drink per person per week, in an average UK household consisting of 2.4 people. There was no evidence of substitution to confectionary or alcohol.

### Strengths and weaknesses

This study used nationally representative data on household purchases collected on a weekly basis over 295 weeks in a large sample. Availability of sociodemographic data enabled us to examine purchases by household income, a commonly used indicator of socioeconomic status,^32^ and presence of children at the household level. However, it was not possible to examine household composition in more granular detail due to limited data availability. The CITS analyses included a non-equivalent control category (toiletries) and at each time point accounted for important factors such as seasonal variations. We also explored the possibility of substitution with other potential sources of sugar (confectionary) and drinks (alcohol). With household purchasing data, it was not possible to record waste or the share of purchases among individuals within a household. The trajectories of the counterfactuals used in the CITS are modelled and based on the trends from March 2014 up until the SDIL announcement (March 2016). However, they may not have continued to take the same course. Attributing changes in the outcomes of interest to the SDIL requires consideration of other events, in particular, the wider UK sugar reduction strategy. However, evidence so far suggests that the strategy has led to minimal changes in purchasing of sugar beyond the effects of the SDIL.^33^

### Comparison with other studies and interpretation of results

SSB taxes target whole populations, but potential health benefits may be greater for some population groups. Here, and in line with some previous studies,^21 22 25^ we observe that low-income households were most responsive to the SDIL. While others have hypothesised that this may be due to greater price sensitivity in lower income households, the SDIL had a complex impact on soft drinks prices.^9^ In addition, we find greater proportional drops in sugar than volume purchased, reinforcing the importance of reformulation alongside any individual level behaviour change. There were also marked differences in baseline purchasing with lower income households purchasing four times as much sugar from soft drinks compared with higher incomes ones. Thus, there may have been more room for lower income households to change their purchasing.

A novel element of our study (particularly timely given recent increases in childhood obesity in UK primary school children during the COVID-19 pandemic^34^) was that households with children were more responsive to the SDIL than those without. Previous work has demonstrated that children are high consumers of SSBs^35^ which, in turn, is associated with childhood obesity.^3^ Furthermore, the SDIL has been associated with a reduction in prevalence of obesity in girls aged 10/11 years in England^36^ and a reduction in childhood hospital admissions for tooth extractions due to caries and asthma.^37 38^ Our findings are compatible with previous studies showing that Mexican households with children reduced sugar from SSBs by 11% following introduction of an SSB tax, compared with only 2% in adult-only households.^21^ As with lower-income households, greater responsiveness among households with children may be due to higher baseline purchasing and differential purchasing of drinks more likely to be reformulated. Furthermore, any signalling effect of the SDIL may have been more salient to households with children, particularly, as it was part of the UK’s Childhood Obesity Plan.^39^

Overall, we found that purchasing of sugar from soft drinks was reduced by 7.5 g (2.6%) per household per week compared with the counterfactual at 19 months, while the volume purchased did not change. While many studies have reported reductions in purchases of taxed SSBs following implementation of taxes, the extent of the reductions differ considerably.^4^ This likely reflects differences in the design of different taxes, differences in baseline consumption and the ease of citizens avoiding a tax by cross-border shopping. Many SSB taxes are intended to increase the price of SSBs relative to non-SSBs. In contrast, the SDIL was primarily intended to incentivise removal of sugar from drinks and this did occur.^9^ The impact of the SDIL on prices was not straightforward, with price revisions across both levied and unlevied drinks.^9^ As the SDIL was implemented nationwide, cross-border shopping is unlikely to be a significant concern. The overall effect size we found is similar to that found in relation to other tiered SSB taxes in Catalonia (2.2% reduction in sugar from SSBs) and in one study in Chile (3.4% in one study,^40^ although 21.6% was reported in another Chilean study^17^).

We find no evidence of a diminishing effect of SDIL on purchased sugar in soft drinks over time. Our findings of an overall reduction in weekly household sugar purchased from soft drinks of 7.5 g (2.9 g, 12.1 g) compared with pre-announcement counterfactuals at 19 months post-implementation are of a similar magnitude to analysis of similar data to 12 months follow-up where a reduction in sugar of 8.0 g (2.4 g, 13.6g) was reported.^8^ This is consistent with findings of a non-diminishing influence of the tax on soft drinks in Mexico at 24 months post-tax implementation. (26,43)

In this study in higher-income households, and those without children, sugar purchased from soft drinks had been consistently falling year-on-year since 2015 but compared with the counterfactual scenario, there was on average a slight increase in sugar purchased from soft drinks. One explanation for this might be the floor effects as these were the groups with the lowest levels of purchasing of sugar from drinks at baseline. The highest income households at baseline for example, purchase about half the amount of sugar from drinks than middle-income households. Since sugar purchasing was already dropping over time, there is likely to have been less scope for the same reductions. It is also possible that these purchasers had preferences for drink products that did not undergo reformulation or already contained less than 5 g sugar per 100 mL and therefore were not reformulated. It has also been observed that when facing government interventions, some households respond counter-intuitively as a form of protest—termed psychological reactance. For instance, reactance was observed immediately following the referendum confirming an SSB tax in Berkeley.^41^ In our study, small increases in purchasing of sugar from soft drinks are noticeable in high-income and middle-income groups (in figure 2b,c) at the time of the SDIL announcement suggesting possible reactance.

Some studies have suggested that price changes in SSBs are linked to changes in purchasing of different alcoholic drinks.^42^ We found little evidence that the SDIL increased purchasing of alcohol—indeed, we found reductions in alcohol purchasing, compared with the counterfactual, in almost all demographic groups. Confectionery purchases remained stable with no evidence of substitution. This is in line with our previous study suggesting soft drinks were not substituted for by confectionery.^8^

## Conclusions and implications for policy and research

Our findings suggest that the impact of the UK SDIL is likely to be greatest in the highest purchasing households (ie, lower income households and those with children). Like other low-agency population interventions, the SDIL has the potential to decrease inequalities in dietary health. We also find persisting effects of the SDIL at 19 months post-implementation on purchasing of sugar from soft drinks, suggesting it may have longer term benefits for population dietary health. After accounting for pre-intervention trends, small increases in sugar purchased from soft drinks were apparent in higher income households and households without children suggesting that a package of different interventions may be required to ensure all members of the population benefit from sugar reduction efforts.

## Supplementary material

10.1136/bmjnph-2024-000981 Supplementary file 1

## Data Availability

No data are available.

## References

[1] Malik VS, Hu FB (2022). The role of sugar-sweetened beverages in the global epidemics of obesity and chronic diseases. Nat Rev Endocrinol.

[2] Han E, Powell LM (2013). Consumption patterns of sugar-sweetened beverages in the United States. J Acad Nutr Diet.

[3] Ooi JY, Sutherland R, Nathan N (2018). A cluster randomised controlled trial of a sugar-sweetened beverage intervention in secondary schools: Pilot study protocol. Nutr Health.

[4] Teng AM, Jones AC, Mizdrak A (2019). Impact of sugar-sweetened beverage taxes on purchases and dietary intake: Systematic review and meta-analysis. Obes Rev.

[5] World Health Organization (2017). Together let’s beat NCDs. Taxes on sugary drinks: why do it?.

[6] GOV.UK (2018). Soft drinks industry levy comes into effect.

[7] Barber S (2017). The soft drinks industry levy.

[8] Rogers NT, Pell D, Mytton OT (2023). Changes in soft drinks purchased by British households associated with the UK soft drinks industry levy: a controlled interrupted time series analysis. BMJ Open.

[9] Scarborough P, Adhikari V, Harrington RA (2020). Impact of the announcement and implementation of the UK Soft Drinks Industry Levy on sugar content, price, product size and number of available soft drinks in the UK, 2015-19: A controlled interrupted time series analysis. PLoS Med.

[10] Hruschka DJ (2012). Do economic constraints on food choice make people fat? A critical review of two hypotheses for the poverty-obesity paradox. Am J Hum Biol.

[11] Lifestyles Team at NHS Digital (2018). Health survey for England 2018.

[12] Briggs ADM, Mytton OT, Kehlbacher A (2013). Overall and income specific effect on prevalence of overweight and obesity of 20% sugar sweetened drink tax in UK: econometric and comparative risk assessment modelling study. BMJ.

[13] Briggs ADM, Mytton OT, Madden D (2013). The potential impact on obesity of a 10% tax on sugar-sweetened beverages in Ireland, an effect assessment modelling study. BMC Public Health.

[14] Schwendicke F, Stolpe M (2017). Taxing sugar-sweetened beverages: impact on overweight and obesity in Germany. BMC Public Health.

[15] Breeze PR, Thomas C, Squires H (2017). Cost-effectiveness of population-based, community, workplace and individual policies for diabetes prevention in the UK. Diabet Med.

[16] Mekonnen TA, Odden MC, Coxson PG (2013). Health benefits of reducing sugar-sweetened beverage intake in high risk populations of California: results from the cardiovascular disease (CVD) policy model. *PLoS One*.

[17] Nakamura R, Mirelman AJ, Cuadrado C (2018). Evaluating the 2014 sugar-sweetened beverage tax in Chile: An observational study in urban areas. PLoS Med.

[18] Fichera E, Mora T, Lopez-Valcarcel BG (2021). How do consumers respond to “sin taxes”? New evidence from a tax on sugary drinks. Soc Sci Med.

[19] Seiler S, Tuchman A, Yao S (2020). The Impact of Soda Taxes: Pass-Through, Tax Avoidance, and Nutritional Effects. Journal of Marketing Research.

[20] Colchero MA, Popkin BM, Rivera JA (2016). Beverage purchases from stores in Mexico under the excise tax on sugar sweetened beverages: observational study. BMJ.

[21] Colchero MA, Molina M, Guerrero-López CM (2017). After Mexico Implemented a Tax, Purchases of Sugar-Sweetened Beverages Decreased and Water Increased: Difference by Place of Residence, Household Composition, and Income Level. *J Nutr*.

[22] Teng A, Buffière B, Genç M (2021). Equity of expenditure changes associated with a sweetened-beverage tax in Tonga: repeated cross-sectional household surveys. BMC Public Health.

[23] Cawley J, Frisvold D, Hill A (2020). Oakland’s sugar-sweetened beverage tax: Impacts on prices, purchases and consumption by adults and children. Econ Hum Biol.

[24] Kaplan S, White JS, Madsen KA (2024). Evaluation of Changes in Prices and Purchases Following Implementation of Sugar-Sweetened Beverage Taxes Across the US. JAMA Health Forum.

[25] Colchero MA, Rivera-Dommarco J, Popkin BM (2017). In Mexico, Evidence Of Sustained Consumer Response Two Years After Implementing A Sugar-Sweetened Beverage Tax. Health Aff (Millwood).

[26] Pedraza LS, Popkin BM, Batis C (2019). The caloric and sugar content of beverages purchased at different store-types changed after the sugary drinks taxation in Mexico. Int J Behav Nutr Phys Act.

[27] Public Health England (2020). Impact of covid-19 pandemic on grocery shopping behaviours.

[28] Office for National Statistics (2020). Proportion of people by annual gross household income band, England, financial year ending 2020.

[29] Office for National Statistics PT (2020). Distribution of non-equivalised household disposable income, financial year ending 2020.

[30] Office for National Statistics (2021). Households by type of household and family, regions of England and UK constituent countries.

[31] White M, Scarborough P, Briggs A Evaluation of the health impacts of the UK treasury soft drinks industry levy (SDIL).

[32] Galobardes B, Shaw M, Lawlor DA (2006). Indicators of socioeconomic position (part 1). J Epidemiol Community Health.

[33] Public Health England (2019). Sugar reduction: report on progress between 2015 and 2018.

[34] Office for Health Improvement and Disparities (2020). NCMP changes in the prevalence of child obesity between 2019 to 2020 and 2020 to 2021.

[35] Dereń K, Weghuber D, Caroli M (2019). Consumption of Sugar-Sweetened Beverages in Paediatric Age: A Position Paper of the European Academy of Paediatrics and the European Childhood Obesity Group. Ann Nutr Metab.

[36] Rogers NT, Cummins S, Forde H (2023). Associations between trajectories of obesity prevalence in English primary school children and the UK soft drinks industry levy: An interrupted time series analysis of surveillance data. PLoS Med.

[37] Rogers NT, Conway DI, Mytton O (2023). Estimated impact of the UK soft drinks industry levy on childhood hospital admissions for carious tooth extractions: interrupted time series analysis. BMJ Nutr Prev Health.

[38] Rogers NT, Cummins S, Jones CP (2024). The UK Soft Drinks Industry Levy and childhood hospital admissions for asthma in England. Nat Commun.

[39] GOV.UK Childhood obesity: a plan for action. https://www.gov.uk/government/publications/childhood-obesity-a-plan-for-action/childhood-obesity-a-plan-for-action.

[40] Caro JC, Corvalán C, Reyes M (2014). Chile’s 2014 sugar-sweetened beverage tax and changes in prices and purchases of sugar-sweetened beverages: An observational study in an urban environment. PLoS Med.

[41] Lee MM, Falbe J, Schillinger D (2019). Sugar-Sweetened Beverage Consumption 3 Years After the Berkeley, California, Sugar-Sweetened Beverage Tax. Am J Public Health.

[42] Quirmbach D, Cornelsen L, Jebb SA (2018). Effect of increasing the price of sugar-sweetened beverages on alcoholic beverage purchases: an economic analysis of sales data. J Epidemiol Community Health.

