# Supplementary material for: Changes in household purchasing of soft drinks following the UK soft drinks industry levy by household income and composition: controlled interrupted time series analysis, March 2014 to November 2019
Source: BMJ Nutr Prev Health. 2025 Jan 16;8(1):e000981. doi: 10.1136/bmjnph-2024-000981 (PMC12322548; doi:10.1136/bmjnph-2024-000981)
Supplement: Supplementary file 1 [file bmjnph-8-1-s001.pdf]

Supplementary Figure legends

Figure S1: Weight (g) of sugar from soft drink products purchased per household per week, by gross household income levels, from March 2014 to November 2019. Observed and modelled amounts of sugar in all soft drinks (drinks liable to the SDIL and non-liable drinks) by annual gross household income levels of a) <20,000 b) £20,000-£50,000 and c) £50,000 or more. Light blue points show observed data and light blue lines (with light blue shadows) shows modelled data (and 95% confidence intervals) of sugar from purchased soft drinks. The dark blue line indicates the counterfactual line based on preannouncement trends and had the announcement and implementation not happened. The first and second dashed lines indicate the announcement and implementation of SDIL, respectively. The red line (and shadow) indicates modelled toiletries (control group). The first and second dashed lines indicate the announcement and implementation of SDIL, respectively.

Figure S2: Weight (g) of sugar from soft drink products purchased per household per week, by whether households have children or not, from March 2014 to November 2019. Observed and modelled amounts of sugar in all soft drinks (drinks liable to the SDIL and non-liable drinks) by a) households with no children b) households with children (<16 years). Light blue points show observed data and light blue lines (with light blue shadows) shows modelled data (and 95% confidence intervals) of sugar from purchased soft drinks. The dark blue line indicates the counterfactual line based on preannouncement trends and had the announcement and implementation not happened. The first and second dashed lines indicate the announcement and implementation of SDIL, respectively. The red line (and shadow) indicates modelled toiletries (control group). The first and second dashed lines indicate the announcement and implementation of SDIL, respectively.

Figure S3: Weight (g) of sugar from confectionery purchased per household per week, by gross household income levels, from March 2014 to November 2019. Observed and modelled amounts of sugar in all soft drinks (drinks liable to the SDIL and non-liable drinks) by annual gross household income levels of a) <20,000 b) £20,000-£50,000 and c) £50,000 or more. Light blue points show observed data and light blue lines (with light blue shadows) shows modelled data (and 95% confidence intervals) of sugar from purchased soft drinks. The dark blue line indicates the counterfactual line based on preannouncement trends and had the announcement and implementation not happened. The first and second dashed lines indicate the announcement and implementation of SDIL, respectively. The scales on the Y axis varies between panels and modelled toiletries have been removed to maximise the resolution of the graphs

Figure S4: Weight (g) of sugar from confectionery purchased per household per week, by whether households have children or not, from March 2014 to November 2019. Observed and modelled amounts of sugar in all soft drinks (drinks liable to the SDIL and non-liable drinks) by a) households with no children b) households with children (<16 years). Light blue points show observed data and light blue lines (with light blue shadows) shows modelled data (and 95% confidence intervals) of sugar from purchased soft drinks. The dark blue line indicates the counterfactual line based on preannouncement trends and had the announcement and implementation not happened. The first and second dashed lines indicate the announcement and implementation of SDIL, respectively. Modelled toiletries have been removed (to maximise the resolution of the graphs.

Figure S5: Volume (mls) of alcohol products purchased per household per week, by gross household income levels, from March 2014 to November. Observed and modelled volumes of alcohol by annual gross household income levels of a) <20,000 b) £20,000-£50,000 and c) £50,000 or more. Light blue points show observed data and light blue lines (with light blue shadows) shows modelled data (and 95% confidence intervals) of volumes of alcohol. The dark blue line indicates the counterfactual line based on preannouncement trends and had the announcement and implementation not happened. The first and second dashed lines indicate the announcement and implementation of SDIL, respectively. The scales on the Y axis varies between panels and modelled toiletries have been removed to maximise the resolution of the graphs

Figure S6: Volume (mls) of alcohol purchased per household per week, by whether households have children or not, from March 2014 to November. Observed and modelled volumes of alcohol by a) households with no children b) households with children (<16 years). Light blue points show observed data and light blue lines (with light blue shadows) shows modelled data (and 95% confidence intervals) of volumes of alcohol. The dark blue line indicates the counterfactual line based on preannouncement trends and had the announcement and implementation not happened. The first and second dashed lines indicate the announcement and implementation of SDIL, respectively. The scales on the Y axis varies between panels and modelled toiletries have been removed to maximise the resolution of the graphs

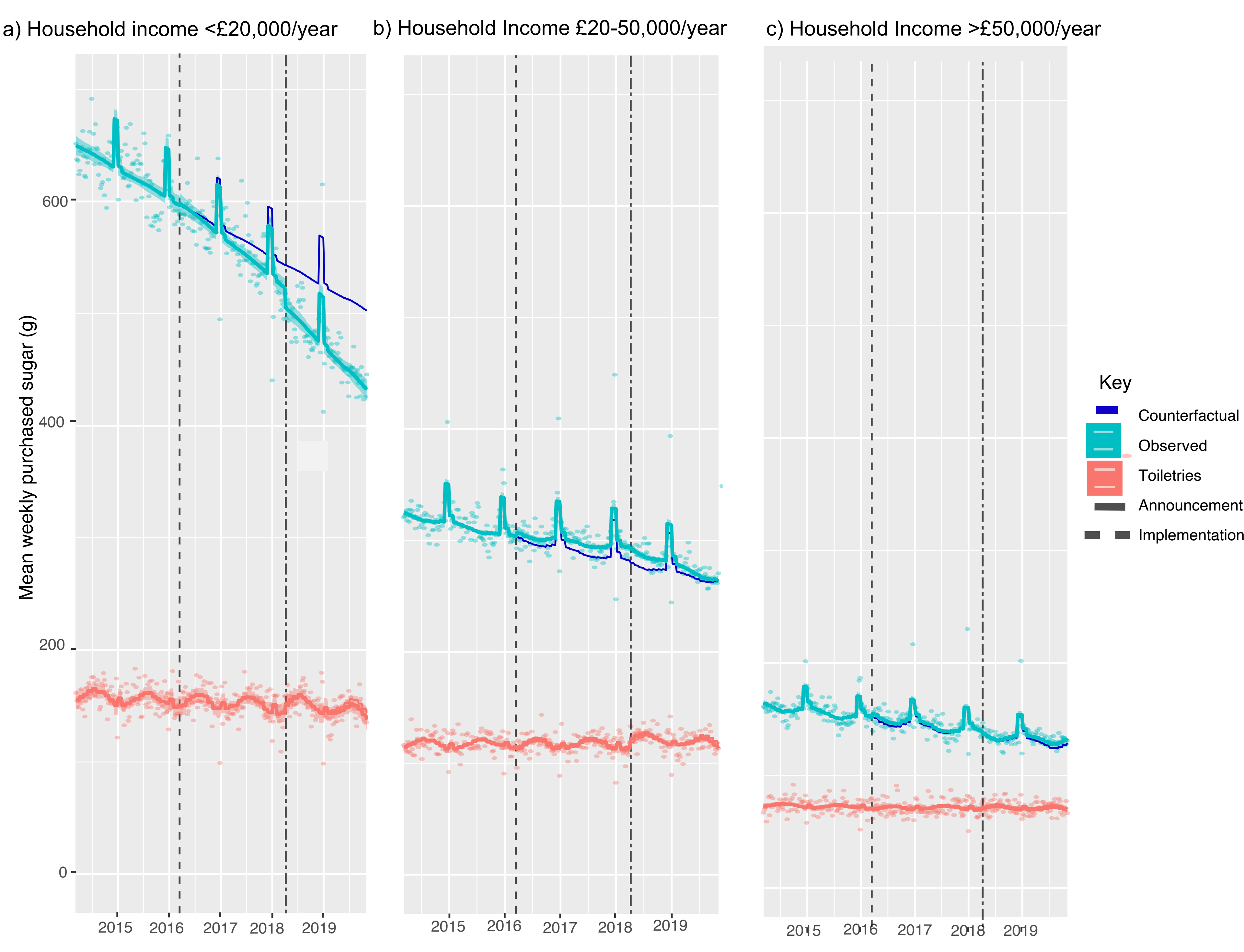

a) Household income <£20,000/year
b) Household Income £20-50,000/year
c) Household Income >£50,000/year
Mean weekly purchased sugar (g)
600
400
200
0
2015
2016
2017
2018
2019
Key
Counterfactual
Observed
Toiletries
Announcement
Implementation

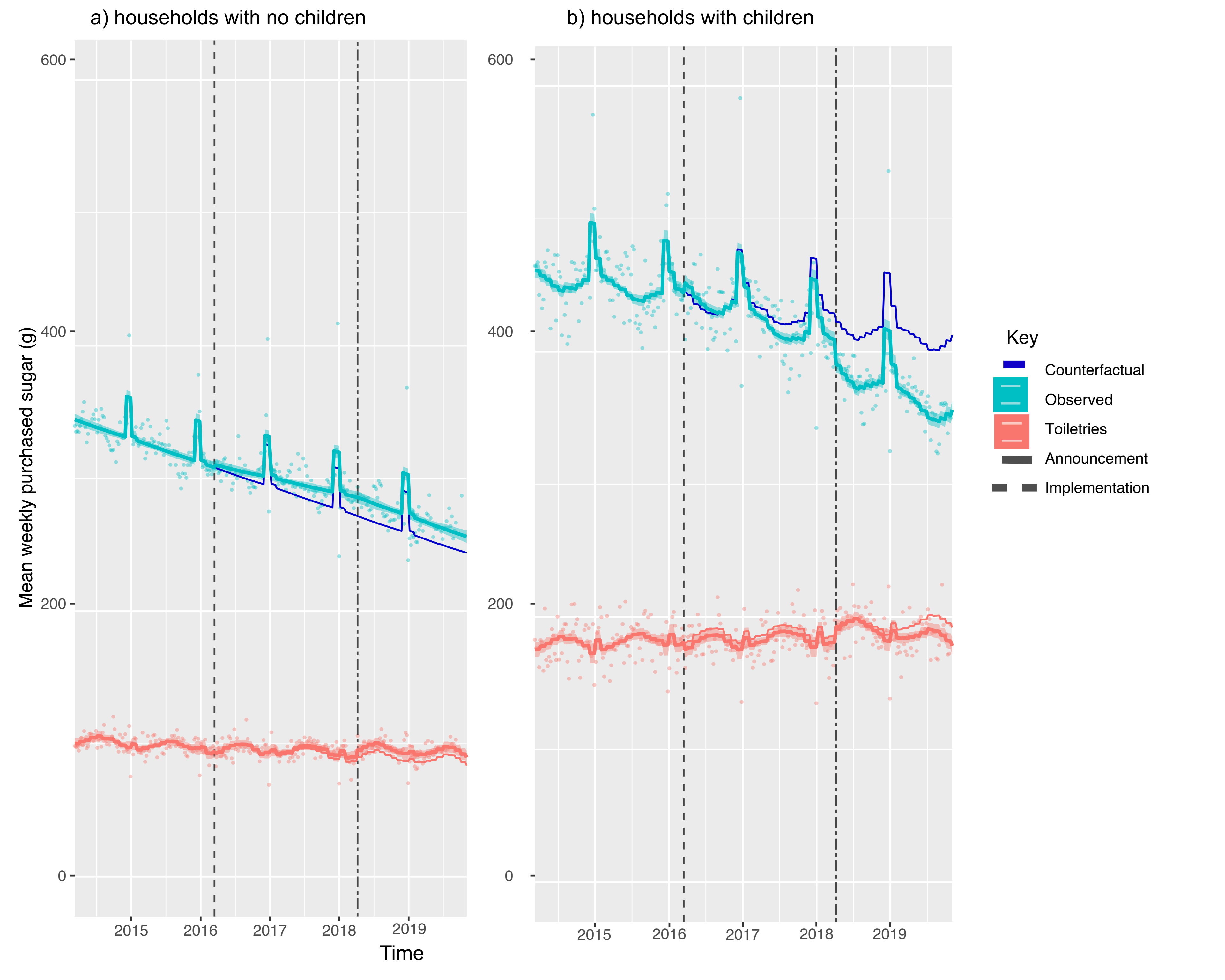

a) households with no children
b) households with children
Mean weekly purchased sugar (g)
600
400
200
0
2015
2016
2017
2018
2019
Time
Key
Counterfactual
Observed
Toiletries
Announcement
Implementation

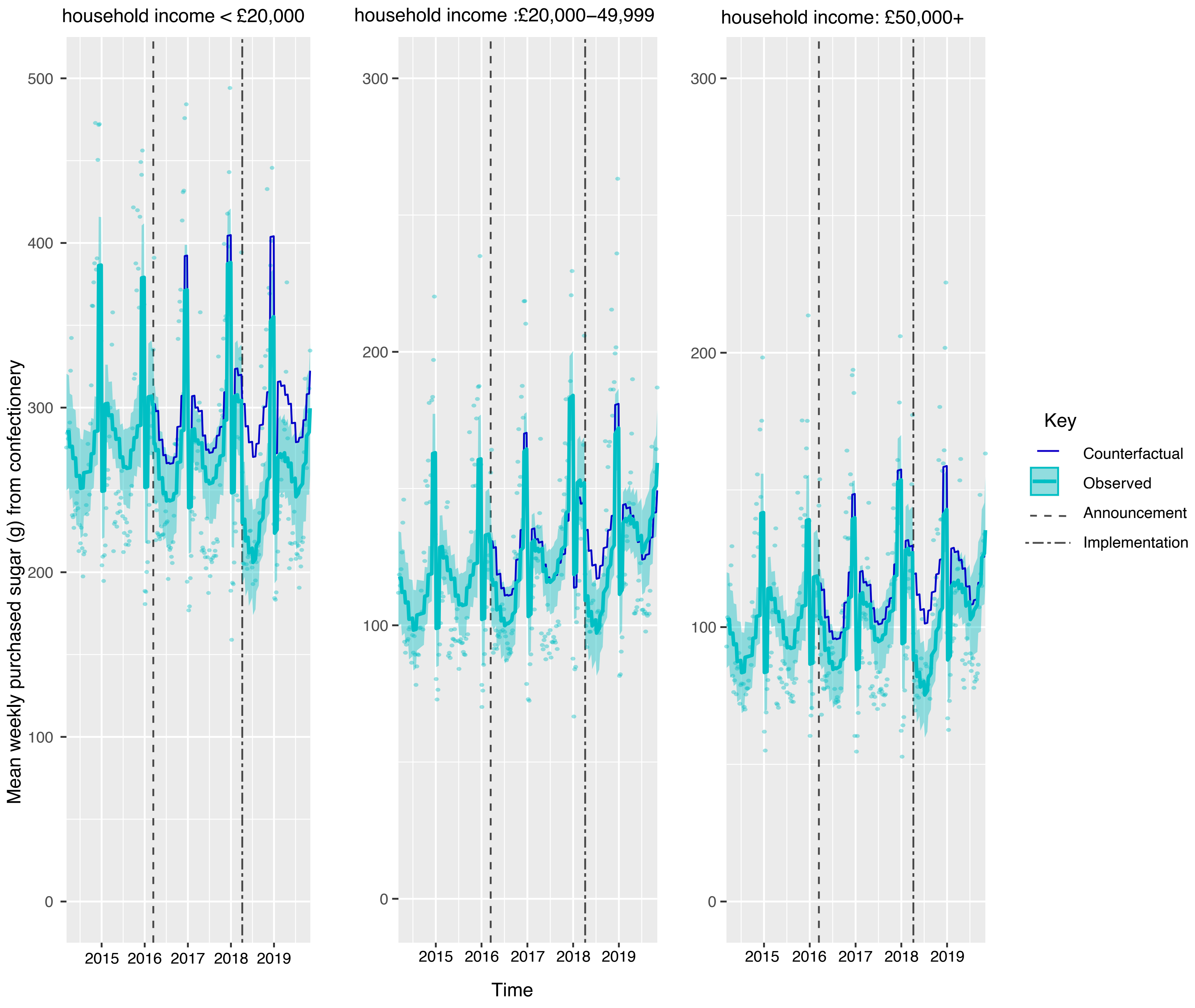

household income < £20,000
household income :£20,000–49,999
household income: £50,000+
Mean weekly purchased sugar (g) from confectionery
500
400
300
200
100
0
2015
2016
2017
2018
2019
Time
Key
Counterfactual
Observed
Announcement
Implementation

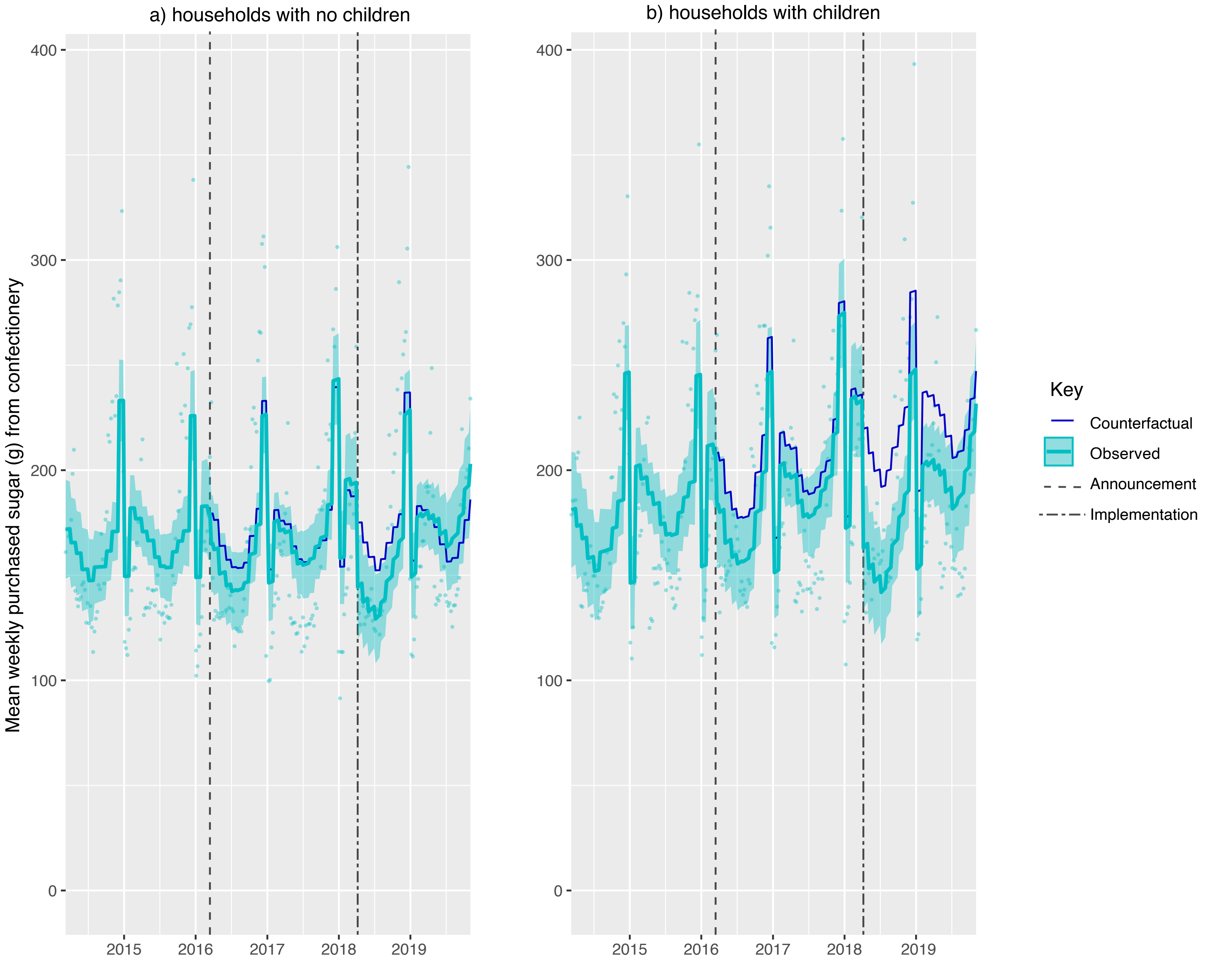
a) households with no children
b) households with children
Mean weekly purchased sugar (g) from confectionery
400
300
200
100
0
2015
2016
2017
2018
2019
Key
Counterfactual
Observed
Announcement
Implementation

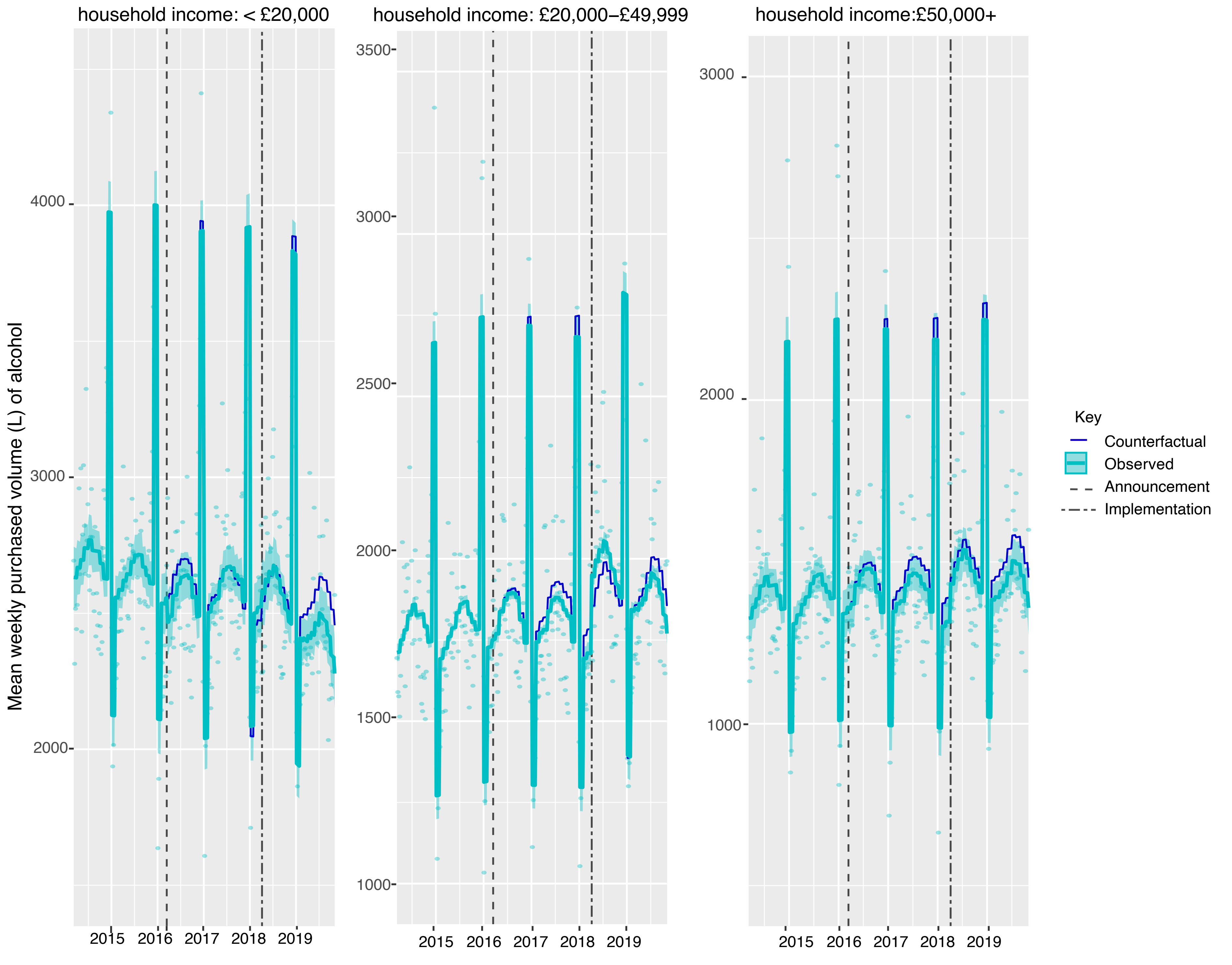
household income: < £20,000
household income: £20,000–£49,999
household income:£50,000+
Mean weekly purchased volume (L) of alcohol
4000
3000
2000
3500
3000
2500
2000
1500
1000
3000
2000
1000
2015
2016
2017
2018
2019
Key
Counterfactual
Observed
Announcement
Implementation

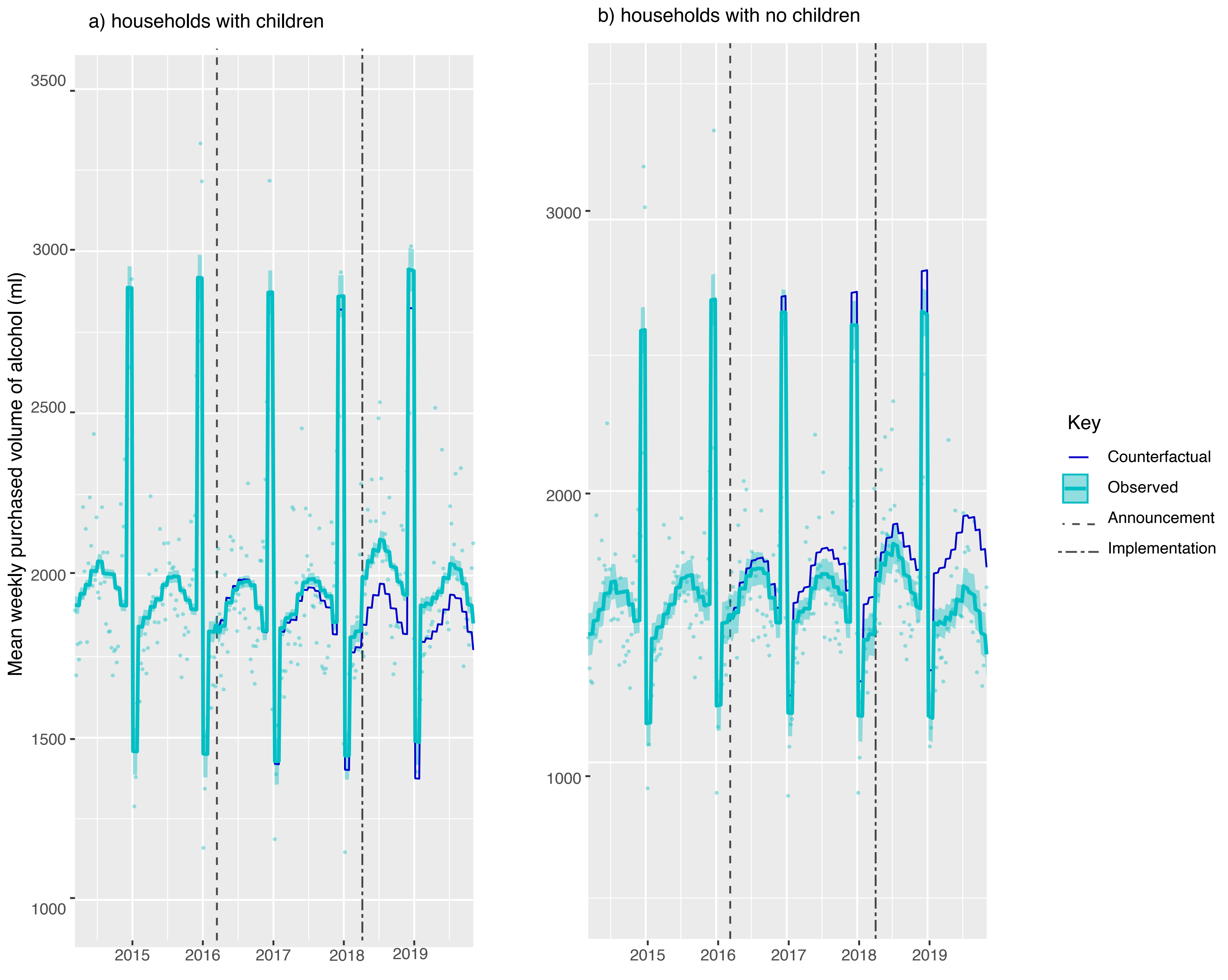

a) households with children
b) households with no children
Mean weekly purchased volume of alcohol (ml)
3500
3000
2500
2000
1500
1000
2015
2016
2017
2018
2019
Key
Counterfactual
Observed
Announcement
Implementation

Table S1: Absolute and relative changes in weight of sugar from confectionery, purchased per household per week compared to the counterfactual estimated from pre-announcement trends, at 19 months post-implementation of the UK soft drinks industry Levy

Sugar (g)	Pre-announcement to post implementation	
	Absolute change (g)	Relative (%)
Total population	6.40(-20.56, 33.36)	3.14(-10.08, 16.36)
Income		
<20,000	-22.8 (-63.02, 17.41)	-7.07(-19.55, 5.40)
20,000-50,000	10.05(-9.56, 29.65)	6.73 (-6.40, 19.86)
>50,000	1.55(-17.94, 21.04)	1.16(-13.41, 15.73)
Children		
Yes	-15.50 (-46.39, 15.39)	-6.27(-18.77, 6.23)
No	16.98(-9.64, 43.59)	9.13(-5.18, 23.43)

Table S2: Absolute and relative changes in volumes from alcohol purchased per household per week compared to the counterfactual estimated from pre-announcement trends, at 19 months post-implementation of the UK soft drinks industry Levy

Volume (mls)	Pre-announcement to post implementation	
	Absolute change (ml)	Relative (%)
Total population[1]	-66.13 (-30.96, -101.31)*	-3.68 (-5.63, -1.72)*
Income		
<20,000	-177.55(-305.58, -49.52)*	-7.23(-12.44, -2.02)*
20,000-50,000	-84.83(-120.54, -49.11)*	-4.57(-6.50, -2.65)*
>50,000	-93.7(-178.3, -9.12)*	-6.45(-12.28, -0.63)*
Children		
Yes	-320.74(-411.22, -230.27)*	-18.66 (-23.93, -13.40)*
No	81.55(45.92, 117.18)*	4.61(2.59, 6.62)*

[1]Whole population includes households with missing values for income (and which represents 8.2% of households) hence total population numbers may not reflect the range of values across income categories.
